# Good practices in central venous catheter maintenance in time of covid-19: an observational study

**DOI:** 10.1590/0034-7167-2021-0397

**Published:** 2022-10-03

**Authors:** Taís Oliveira Dias, Luciana Guimarães Assad, Vanessa Galdino de Paula, Luana Ferreira de Almeida, Erica Brandão de Moraes, Pedro Ruiz Barbosa Nassar

**Affiliations:** I Universidade do Estado do Rio de Janeiro. Rio de Janeiro, Rio de Janeiro, Brazil; II Universidade Federal Fluminense. Niterói, Rio de Janeiro, Brazil

**Keywords:** Critical Care Nursing, Patient Safety, Catheter-Related Infections, Coronavirus Infections, Hospital Infection Control Program., Enfermería de Cuidados Críticos, Seguridad del Paciente, Infecciones Relacionadas con Catéteres, Infecciones por Coronavirus, Programa de Control de Infecciones Hospitalarias., Enfermagem de Cuidados Críticos, Segurança do Paciente, Infecções Relacionadas a Cateter, Infecções por Coronavírus, Programa de Controle de Infecção Hospitalar.

## Abstract

**Objectives:**

to assess adherence to good practices for central venous catheter maintenance by the nursing team during the COVID-19 pandemic.

**Methods:**

observational, cross-sectional, quantitative research with non-participant observation. Data collection was guided by an instrument developed for this study, consisting of five dimensions. It took place in the intensive care unit of a university hospital in the city of Rio de Janeiro.

**Results:**

a total of 700 observations were carried out, which resulted, in general, in 402 (57.4%) procedures for adherence to good practices. Hand hygiene (8%) and Performing the dressings (10%) were the dimensions with the lowest adherence.

**Conclusions:**

good practices for central venous catheter maintenance were partially present in the routine of the nursing team during the COVID-19 pandemic. In critical moments, intensifying the qualification of the teams for a better adaptation to the new work processes is a strategy to sustain the patient safety culture.

## RESUMO

**Objetivos:**

avaliar a adesão às boas práticas de manutenção do cateter venoso central pela equipe de enfermagem durante a pandemia de COVID-19.

**Métodos:**

pesquisa observacional, transversal, quantitativa com observação não participante. A coleta de dados foi orientada por instrumento desenvolvido para este estudo, constituído por cinco dimensões. Ocorreu em unidade de terapia intensiva de um hospital universitário na cidade do Rio de Janeiro.

**Resultados:**

foram realizadas 700 observações, que resultaram, no geral, em 402 (57,4%) procedimentos de adesão às boas práticas. Higiene das mãos (8%) e Realização de curativos (10%) foram as dimensões com menor adesão.

**Conclusões:**

as boas práticas de manutenção do cateter venoso central mostraram-se parcialmente presentes na rotina da equipe de enfermagem durante a pandemia de COVID-19. Em momentos críticos, intensificar a qualificação das equipes para uma melhor adaptação aos novos processos de trabalho é uma estratégia para sustentar a cultura de segurança do paciente.

## RESUMEN

**Objetivos:**

evaluar adhesión a buenas prácticas de manutención del catéter venoso central por grupo de enfermería en tiempos de COVID-19.

**Métodos:**

investigación observacional, transversal, cuantitativa con observación no participante. La recolecta de datos fue orientada por instrumento desarrollado para este estudio, constituido por cinco dimensiones. Ocurrió en unidad de terapia intensiva de un hospital universitario en Rio de Janeiro.

**Resultados:**

fueron realizadas 700 observaciones, que resultaron, en el general, en 402 (57,4%) procedimientos de adhesión a las buenas prácticas. Higiene de las manos (8%) y Realización de curativos (10%) fueron las dimensiones con menor adhesión.

**Conclusiones:**

las buenas prácticas de manutención del catéter venoso central se mostraron parcialmente presentes en la rutina del grupo de enfermería durante la pandemia de COVID-19. En momentos críticos, intensificar la calificación de los grupos para una mejor adaptación a los nuevos procesos laborales es una estrategia para sustentar la cultura de seguridad del paciente.

## INTRODUCTION

The public health emergency caused by the COVID-19 pandemic has brought numerous challenges and changes to the work of the nursing team, which works on the front line of care for these patients. In the hospital setting, measures for infection prevention and control, such as practices that involve safe maintenance of short-term central venous catheters (CVC), have become even more complex^(1-2)^.

This device is essential in intensive care units (ICU), especially for patients with COVID-19 who have a rapid, severe evolution and prolonged hospitalization^(1,3-4)^. The CVC enables rapid infusions of volumes and drugs that cannot be administered in peripheral vascular accesses, provides continuous hemodynamic monitoring when connected to a monitor, possibility of infusion of parenteral nutrition, in addition to being a route for venous blood collection^(3,5)^.

The long stay of these patients demands meticulous care in handling accesses, in order to avoid possible adverse events such as healthcare-associated infections (HAI). HAIs are considered the fourth cause of complications in developed countries and have a great epidemiological impact, increasing institutional costs, length of stay and the morbidity and mortality rate^(6-7)^.

Among the prevalent causes of HAIs, the use of invasive devices is pointed out as one of the main factors that influence their emergence. In the case of catheter-related bloodstream infections (CRBSI), the use of CVC is responsible for approximately 90% of ICU infections^(3)^. In 2018, 27,957 cases were reported in Brazilian hospitals, with an incidence density in adult ICUs of 4.1 cases per thousand catheter-days, and the estimated limit for a critical unit is values up to 0.5-1 per thousand catheter-days^(8-9)^.

In 2020, the Brazilian association of intensive medicine (AMIB) published a scale of priorities regarding the care of adult patients with COVID-19 and highlighted the management of venous catheters. The classification reinforces that measures to prevent CRBSI are extremely important, need to be performed in the nurse’s daily routine and should only be practiced in a secondary way when the patient’s emergency needs are a priority^(10)^.

It is understood that dealing with the lack of knowledge of the new virus, with fear, anxiety, illness of team members, new routines, and work processes, in addition to exhausting journeys using personal protective equipment, was a huge challenge for the team. As a result, there were high numbers of absenteeism, constant reassignments, successive temporary hires and a greater risk of incidents that could harm patient safety^(2,10)^.

In addition, the team had to reorganize itself to care for prone patients, which implied greater difficulties in performing CVC dressings and administering medication. Monitoring the catheter insertion site became difficult; disconnections of infusion lines and contamination of the circuit with patient secretions could not be visualized; and those inserted into jugular and subclavian veins caused greater exposure of the professional, given the closer manipulation of the airways, which is a site of greater transmissibility and spread of the virus, by the generation of droplets and/or aerosols^(1)^.

This new reality presented itself as a risk factor for the safety of health professionals and patients. In this sense, the need to continue with the continuous evaluation of the practices adopted for the safe maintenance of the CVC emerged, to maintain excellence in good practices, reduce incidents and subsidize the offer of new training during the pandemic. Thus, the following research problem was formulated: Are good CVC maintenance practices present in the routine of the nursing team during the COVID-19 pandemic?

## OBJECTIVES

To assess adherence to good practices in central venous catheter maintenance by the nursing team during the COVID-19 pandemic.

## METHODS

### Ethical aspects

The research project was analyzed and approved by the Research Ethics Committee, meeting the ethical principles required by Resolution 466 of 2012, of the National Health Council^(11)^.

### Study design, period, and location

This is an observational, cross-sectional, quantitative research based on non-participant observation carried out from April to May 2020. It was carried out in the ICU of a university hospital (in the city of Rio de Janeiro, Brazil) that contains ten hospital beds for adults, directed, during the time of a pandemic, to the critical care of patients diagnosed with COVID-19. For the design, conduct and reporting of the study, the Strengthening the Reporting of Observational Studies in Epidemiology (STROBE) initiative was adopted^(12)^.

### Population

Nurses, residents, and nursing technicians participated in the research. Professionals who handled the CVC of patients in that unit were included and those who were on vacation or leave during the collection were excluded. The population of employees described in the work schedules was represented by 65 professionals who worked in the unit during the studied period, among which 15 nurses, 7 residents and 43 nursing technicians, who were observed in all maintenance steps and obtained the same opportunities during observation.

All invited participants received the Free and Informed Consent Form (ICF) and were oriented in relation to the research objectives, its voluntary nature, in addition to having the opportunity to clear existing doubts. After the participant’s acceptance and signature, a copy of the form was delivered to him, making it possible to start the observations.

### Study protocol

Data collection was performed by convenience sampling of procedures related to CVC maintenance, without predetermination or professional choice. It was developed from April to May 2020 and conducted by the lead author of the study.

Considering the lack of studies that address the result of health professionals’ adherence to the recommendations of good practices for infection prevention and the need to choose strategies for their development^(13)^, observation was selected as a technique for the development of the collection of data.

The observations were guided by a structured, non-validated form, developed for this study and based on the Bloodstream Infection Prevention Measures^(14)^ and the Guideline for the Prevention of Intravascular Catheter-Related Infections^(15)^. The final version contained 5 dimensions and 14 items, as described in Chart 1.

**Chart 1:** Instrument for collecting data on adherence of the nursing team to good practices in central venous catheter maintenance, Rio de Janeiro, Rio de Janeiro, Brazil, 2020

Dimension 1: Hand hygiene
1. Hygienize your hands correctly with soap and water for 40 to 60 seconds or with an alcohol-based preparation for 20 to 30 seconds before handling the catheter.
**Dimension 2: Personal protective equipment**
2. Use personal protective equipment (mask, cap, glasses, and gloves) whenever handling the catheter.
**Dimension 3: Scrub the hub**
3. Scrub the hub with movements to generate mechanical friction, from 5 to 15 seconds before accessing the catheters, connectors, and side ejectors.
4. Scrub the hub with 0.5% chlorhexidine solution or 70% alcohol, waiting for it to dry.
**Dimension 4: Performing the dressings**
5. Use sterile gloves.
6. Use 0.5% alcoholic chlorhexidine solution for antisepsis.
7. Perform only unidirectional movements; always from the ostium to the distal part.
8. Wait for the applied solution to dry spontaneously.
9. Use sterile gauze or sterile transparent dressing as a cover.
10. Change the dressing every 24 hours when sterile gauze is used, or every seven days when the sterile transparent dressing is installed.
**Dimension 5: Records**
11. Record the date of exchange of equipment (in medical records, nursing form or on the device, valid for up to 96 hours).
12. Record the connector replacement date (in medical records, nursing form or on the device, valid for up to 96 hours).
13. Record the dressing change (date and signature of the professional who performed it) in the medical record, nursing form or on the dressing cover.
14. Record the presence of phlogistic signs (date and signature of the professional who made the observation) in the medical chart or nursing form.

Surveys carried out at the study locus estimate that, on average, one hundred procedures are performed per day to maintain the central venous catheter of hospitalized patients, including the entire list of practices analyzed in this research. Since the seven-day period includes the work rotation of all health professionals participating in the study, it was established as necessary to observe the performance of 700 procedures. Considering the inclusion of 14 items in the instrument, there are 50 observations regarding the performance of each one.

To mitigate changes in the practice and behavior of professionals during observation and maintain their spontaneity, some strategies were outlined, such as the prior delivery of the Free and Informed Consent Form (ICF) and care with the observer’s positioning, keeping themself in the position of the nurse - this allowed a broad view of all beds and a certain distance from the professionals, so that the collaborator did not associate the presence of the observer with an evaluation. This strategy was made possible because the researcher was an integral part of the team, which justified her presence in the room in addition to the observation of the professionals.

The term “adherence” was adopted when all items referring to the dimension were met; and the term “non-adherence” in the situation in which at least one item of the dimension was not in compliance. The frequencies of compliance and non-compliance were then calculated for all dimensions and items of the data collection instrument.

### Analysis of results and statistics

For data collection and storage, the Google Forms platform was used, with subsequent transfer and processing in Microsoft Excel 2010. Then, the data were exported to the Stata v.15 software for descriptive and inferential analysis. In the inferential analysis, Fisher’s exact and chi-square tests were applied to verify the presence of an association between compliance performance and professional category (nurse, resident, and nursing technician).

Subsequently, in order to correlate the performance in the dimensions, scores were assigned to the items and dimensions. Items with adherence received 1 point, and those without adherence received a score of 0. The final score of the dimension was represented by the sum of its items. Spearman’s correlation tests were used to verify the relationship between scores of the dimensions on CVC maintenance. It was determined: null correlation, 0.00; very weak, 0.01 to 0.29; moderate, 0.30 to 0.59; very high, 0.80 to 0.99; and perfect, 1.00. The significance level adopted throughout the study was 5%.

## RESULTS

Fifty observations were made of each of the 14 items of the instrument, which totaled 700 observed procedures related to CVC maintenance practices. It was found, in general, that 402 (57.4%) procedures were adhered to by the team, and 298 (42.6%) were not adhered to. In Table 1, one can observe the rates of adherence to CVC maintenance practices according to the dimensions and items observed.

**Table 1:** Adherence to good practices for central venous catheter maintenance by members of the nursing team, Rio de Janeiro, Rio de Janeiro, Brazil, 2020 (n = 50)

Dimensions	n	%
Hand hygiene	4	8
Soap and water (40 to 60 s) or alcohol preparation (20 to 30 s)	4	8
Personal protection equipment	47	94
Use of mask, cap, goggles, and gloves	47	94
Scrub the hub	23	46
Disinfection with 70% alcohol for 5 to 15 seconds, waiting for drying	11	22
Disinfection with 0.5% chlorhexidine for 5 to 15 seconds, waiting for drying	12	24
Performing the dressings^*^		
Use sterile glove	44	88
Antisepsis of the puncture site with 0.5% chlorhexidine	43	86
Perform antisepsis with unidirectional movements	22	44
Allow the applied solution to dry spontaneously.	10	20
Maintain sterile coverage with gauze or clear film	49	98
Perform exchange according to routine	50	100
Records^*^		
Connector replacement	0	0
Change of equipment	19	38
Phlogistic signs	32	64
Dressing change	36	72

* *In the dimension “Records” and “the Performing the dressings”, their final sum was not presented, as the items were evaluated separately as they are not mutually exclusive.*

Hand hygiene was the dimension with the lowest adherence to good practices (8%), that is, in 92% of the observations, professionals did not perform it or did not attend to its phases and time effectively. They did not incorporate any of the parts such as the palms of the hands, between the fingers, back of the fingers, thumb, digital and wrists, with damage to the adequate time.

The majority (94%) of the professionals used all personal protective equipment such as a mask, cap, glasses, and gloves during catheter manipulations. In addition to these, during the collection period, all employees adhered to the use of waterproof apron, N95/PFF2 mask and face shield type face shield, equipment indicated for aerosol precautions.

Adherence to the extent of performing the scrub the hub was 46%, that is, most of the team did not disinfect the devices before accessing the venous line. When performed, there was a balance in the choice of solution between alcohol (22%) and alcoholic chlorhexidine (24%).

As for the dressing of the CVC, 88% of the observations were performed using sterile gloves, and the 12% who did not adhere to this practice were those who, when performing the dressing at another puncture site of the same patient, did not change the gloves. Puncture site antisepsis with 0.5% chlorhexidine was met in most observations (86%), however adherence to antisepsis with unidirectional movements was low (44%) and even lower was spontaneous drying (20%). The routine dressing change procedure was met (100%), and the use of gauze or film coverage was 98%.

Regarding the written communication of nursing information relevant to the client and the care received by the team, the records of phlogistic signs (64%) and the change of dressings (72%) were the notes with the highest adherence in the observations, while the exchange of equipment had low adherence (38%) of professionals, and no record of exchange of connectors was found.

As shown in Table 2, nurses showed greater adherence in the set of dimensions related to CVC maintenance, while nursing technicians had the lowest adherence in all items of good practices in which they were observed. It was identified that the professional category is associated (p < 0.05) with performance in three dimensions of good practices in maintaining central venous access.

**Table 2:** Association between adherence to good practices in maintaining central venous access and professional category, Rio de Janeiro, Rio de Janeiro, Brazil, 2020

Dimensions	Nurses%	Residents%	Technician%	*p* value
Hand hygiene	40	6.6	3.3	< 0.019^†^
Personal protection equipment	100	100	89.6	0.315^‖^
Scrub the hub	100	55.2	26.3	0.043^‖^
Performing the dressings	60	10	2.8	< 0.001^†^

*† Fisher’s Exact Test;* ‖ *Pearson’s chi-square test. The “Records” dimension was not included, as it was not possible to identify the professional category in all records.*

In Spearman’s correlation analysis, shown in Table 3, there was a statistically significant result in the Scrub the hub (3) and Records (5) dimensions, with a negative correlation coefficient (-0.359), that is, when the score of one increases the other decreases. Furthermore, PPE (2) and Scrub the hub (3) showed a significant correlation coefficient, however of weak magnitude (0.233).

**Table 3:** Correlation between scores of dimensions on maintenance of central venous access, Rio de Janeiro, Rio de Janeiro, Brazil, 2020

	Dimension 1	Dimension 2	Dimension 3	Dimension 4	Dimension 5
Dimension 1	1.00				
Dimension 2	0.074 (0.60)	1.00			
Dimension 3	0.023 (0.87)	0.233 (0.01)	1.00		
Dimension 4	-0.098 (0.49)	0.084 (0.56)	-0.173 (0.22)	1.00	
Dimension 5	0.121 (0.48)	-0.097 (0.57)	-0.359 (0.03)	-0.071 (0.68)	1.00

*Spearman correlation. Correlation coefficient (p value); bold = p < 0.05; Dimension 1 - Hand hygiene; Dimension 2 - Personal protective equipment; Dimension 3 - Scrub the hub; Dimension 4 - The dressing; Dimension 5 - Records.*

## DISCUSSION

CRBSI is a potentially preventable adverse event, as it represents the occurrence of harm to the patient and is associated with an active failure or a latent condition, or even a violation of norms and standards. Monitoring this event is considered an indicator of the quality of care that reflects the condition of the care provided and indicates compliance or not with criteria related to patient safety^(13-14)^.

Faced with the pandemic caused by the new coronavirus, SARS-CoV-2, hospitals had to reorganize their work processes to meet this demand, and professionals adapted to a new model of care in the face of a still unknown disease. This study sought to monitor adherence to good practices for central venous catheter maintenance in an intensive care unit of a university hospital that changed its profile to assist patients with COVID-19.

In a study on the assessment of adherence by the multi-professional team to measures to prevent CVC-related bloodstream infection, it was observed that nursing professionals neglected basic measures that directly influenced the prevention of these infections, exposing both patients and professionals to the risk of contamination^(16)^. By correlating these findings with the results presented in this research, it was possible to show that the nursing team, by not fully adhering to the central venous catheter maintenance protocol, put their safety and that of the patient at risk in terms of infection.

Despite simple actions, known and proven worldwide as effective, it is perceived that the knowledge and behavior of professionals still have divergences, impairing adherence to good practices^(5)^. Hence, the importance of studies that point out these results and provide subsidies for the construction of strategies aimed at overcoming these barriers.

Although most procedures were in accordance with the recommendations, the number of non-compliances, even when small, must be considered, as it impacts infection control, quality of care and patient safety. In this study, the culture of zero tolerance to infections is defended; and, for that, it is necessary to work with adhesion goals of the teams that get closer and closer to 100% adhesion. For bundles to be effective in their proposal, studies defend the need for a high rate of team adherence; and, even with a satisfactory global result, educational interventions and monitoring of adherence rates are necessary to promote an adequacy between knowledge and improvement of practice^(3,16)^.

Among the procedures, the ones that showed less adherence by the team were hand hygiene and the dressing, considered potential indicators for the emergence of CRBSI. When associating the professional nursing categories, it was observed that nurses adhered more to good practices in all procedures observed. Nursing technicians as well as residents showed low compliance in all dimensions and especially in hand hygiene, which impacted the reduced result regarding adherence to this dimension.

Established by the World Health Organization as the number 5 international patient safety goal, hand hygiene is one of the main measures to prevent HAIs. It is a practice recognized as the first choice in infection control for being simple, effective and low cost; however, its adherence is still considered a difficulty and a challenge for managers and health institutions, especially in developing countries, whose infection rate can be up to 20 times higher than in developed countries^(17)^.

It has been shown that the use of gloves is directly related to the low frequency of hand hygiene among health professionals^(16)^. In the specific case of this study, this relationship was confirmed, since, as professionals needed continuous attire inside the COVID ICU and gloves were one of the main protective barriers used to avoid direct contact with the patient, hand hygiene was less frequent. It was observed that many professionals put on several gloves and removed them one at a time after each procedure performed, or they cleaned the gloves with 70% alcohol. This practice is not recommended for health care, and it is recommended that hand hygiene be performed before any contact or procedure with the patient, and only afterwards, gloves should be worn. Its removal must be immediate and discarded as infectious waste, followed by new hand hygiene^(18)^.

Associated with hand hygiene and to create a barrier to the spread of infections related to the use of CVC, care with the hub is highlighted, considering that microorganisms present in it can migrate to the internal lumen of the catheter and cause current infection. blood. Thus, disinfection with 70% alcohol or 0.5% alcoholic chlorhexidine for 5 to 15 seconds is recommended before medication administration^(14)^. Adherence to the Scrub the Hub dimension did not fully meet the best practice recommendation. The result is in line with other research, which led to the creation of the “Scrub the Hub” campaign by the Association for Professionals in Infection Control and Epidemiology, with the aim of including this recommendation in CVC training and maintenance protocols and consequently reducing HAIs^(15)^.

With regard to dressing the CVC, the nursing team demonstrated that it did not fully adhere to good practices in this dimension. This was because, despite the fact that most professionals applied the dressing according to the recommendations, there was low adherence to the spontaneous drying time of the solution and to the performance of antisepsis with unidirectional movements. The insecurity when dealing with an as yet unknown disease, the fear of being contaminated with the patient’s oral and tracheal secretions while dressing the accesses close to this region, such as the subclavian and jugular veins, and the difficulties inherent in patient care prone for up to 16 hours may have affected this item’s result. These new conditions created difficulties for the team to develop the correct technique during this procedure; however, it is noteworthy that it is mandatory, in any condition, to perform a dressing with aseptic technique in deep venous accesses, in addition to changing it in the presence of dirt, loosening or moisture^(14)^.

It was found that the use of personal protective equipment was the dimension that obtained the greatest adherence by professionals. This reality is associated with the requirement of attire for the care of every patient affected by the new coronavirus; and was fully met by the team as a safety barrier in the provision of care to patients affected by a serious and not yet fully known disease.

As for the record, the items related to the exchange of connectors and equipment had the lowest adherence scores. Although the study scenario had exchange rules according to the standards recommended by the Hospital Infection Control Commission and this routine was met during observation, there was no note of this procedure in the patient’s device, in the medical record or in the nursing form.

One of the work processes changed during the pandemic was the reorganization of access to medical records for recording, to minimize the risks of contamination of professionals with fomites; thus, the printed and physical records were removed from the scenario of direct patient care, which made it even more difficult to carry out the records. The conducts and procedures performed must always be recorded in medical records or nursing forms, an essential condition to guarantee the excellence of the care process, as effective communication between the teams ensures the quality and safety of the patient, serves as legal evidence and meets the processes of audits, which generates decision-making^(19)^.

The CRBSI has great preventive potential: it is estimated that 70% of cases could be avoided just by adhering to bundles of good practices^(12)^. It is believed that the pandemic may have been a major risk factor for the increased number of non-compliances in the dimensions observed in this study.

It is essential to standardize and develop strategies that allow increasing adherence to protocols for CVC maintenance, in order to guide the decisions and conduct of professionals during care^(9)^. This process should be used not only to correct non-conformities, but especially during times of changes in work routines and processes, to maintain quality in patient care and safety.

Strategies such as the implementation of care bundles, the offer of continuing education, the transparency of communications and the monitoring of the teams are measures that have been discussed as important for the reduction of CRBSI in health institutions, considering their impact on the adherence of workers to measures of good care practices^(16,20)^.

The results presented in this research are not sufficient to assess the emergence of CRBSI, however the study was important for monitoring adherence to good CVC maintenance practices, considering the reorganization of teams in view of the variability of work processes. For the control of CRBSIs, it is necessary to associate process indicators, audits and epidemiological surveillance carried out by the institution. In addition, the results of blood cultures, choice of site and techniques used during CVC insertion and antiseptic drug preparation are necessary for the final diagnosis of the evaluations.

### Study limitations

In view of the specificity of the pandemic moment, it was noticed that there was not enough time for publications that involved the monitoring of good practices in the maintenance of CVC in patients with COVID-19, which determined a lack of reference on the specific theme of this study. There was also a limitation regarding the availability of studies that allowed establishing strata consisting of percentages of adherence based on the adequate performance of CVC maintenance practices.

In addition, it was also limited by the fact that the research was carried out in a single unit of the institution, due to the institutional norms of the moment, which demanded the reduction of the movement of employees between sectors and the allocation of professionals only in their unit of origin, which prevented the researcher from being present in other ICUs.

### Contributions to the area of Nursing, Health, or Public Policy

As it was developed when the institution was reorganizing itself to meet the demand of patients affected by COVID-19, the research supported nursing managers of the institution’s ICUs for decision-making in maintaining the patient safety culture during profound changes in work processes.

The study allowed continuity through the development of other specific research in the ICUs of the study scenario, with the offer of continuing education and a reflective practice for the team with the objective of better adherence to CVC maintenance bundles.

As part of the Research Group - Health and Nursing Technology in the Context of Patient Safety in a Hospital Environment, the study contributed to the advancement of knowledge production in the area of patient safety and strengthening of a group that involves teachers, nurses and lato and strictu sensu undergraduate and graduate students.

In order to complement this research, we encourage the development of new works that address infection control and its relationship with good practices during CVC insertion; offering and validating educational strategies for greater adherence to the CVC maintenance bundle; monitoring related to the antiseptic preparation of medicines during the care of patients affected by the new coronavirus; as well as research that allows an analysis of the gaps between theoretical knowledge and its applicability in the practice of care.

## CONCLUSIONS

Good CVC maintenance practices were partially present in the routine of the nursing team during the COVID-19 pandemic. The study showed good adherence in the use of PPE, while hand hygiene, dressing, scrub the hub and records proved to be practices that need intervention for better adherence.

The search for full adherence to good practices in CVC maintenance should be a goal for team leaders and managers of highly complex units, especially in times of need to adjust work processes, as was the case during the COVID-19 pandemic. Monitoring these practices makes it possible to identify the need to implement strategies, including professional education policies, which can be extended to other health contexts.

During moments of crisis such as facing a pandemic, qualifying the team to adhere to good practices is intended to prevent the emergence of injuries generated by the ICSRC and to improve the comfort and safety of patients and professionals, when all procedures are carried out correctly, continuously, and simultaneously.

In addition, other permanent strategies to improve adherence to meet patient safety are pointed out. The greater involvement of senior management to consolidate patient safety as an institutional policy and the training of leaders capable of acting on any necessary change in work processes so as not to have a negative impact on the reorganization of health care can be mentioned.

It is also important to encourage a culture of group work and the formation of multidisciplinary teams and leaders that help to maintain a climate of collaboration in such a way that everyone can reflect together on necessary changes and suggest improvements.

Finally, the maintenance of the strategy of observation of practices or the implementation of periodic clinical audits is encouraged with a survey of quality indicators that guide the decision-making of managers and contribute to the development of best practices in health care.
